# Sorption of Nickel(II) on a Calcareous Aridisol Soil, China: Batch, XPS, and EXAFS Spectroscopic Investigations

**DOI:** 10.1038/srep46744

**Published:** 2017-04-25

**Authors:** Shirong Qiang, Bin Han, Xiaolan Zhao, Yunbo Yang, Dadong Shao, Ping Li, Jianjun Liang, Qiaohui Fan

**Affiliations:** 1 Key Laboratory of Petroleum Resources, Gansu Province/CAS Key Laboratory of Petroleum Resources Research, Institute of Geology and Geophysics, Chinese Academy of Sciences, Lanzhou, Gansu, 730000, China; 2 Key Laboratory of Preclinical Study for New Drugs of Gansu Province, and Institute of Physiology, School of Basic Medical Sciences, Lanzhou University, 199 Donggang West Road, Lanzhou 73000, China; 3 Graduate University of Chinese Academy of Sciences, Beijing, 100049, China; 4 Institute of Plasma Physics, Chinese Academy of Sciences, Hefei 230031, China

## Abstract

The sorption of Ni(II) on a calcareous aridisol (CA) soil, one of the major soil types in northwestern China, was investigated using batch and extended X-ray absorption fine structure (EXAFS) approaches in a 0.01 mol/L NaClO_4_ solution at different pH values (6.0–10.0), temperatures (25–60 °C) and contact times (2–15 days). Under alkaline conditions, EXAFS analysis showed that the interatomic distances between Ni and O atoms (*R*_Ni-O_) were approximately 2.04 Å with a typical coordination number (*CN*) of ~6.0 O atoms in the contact time range from 2 to 15 days. The *R*_Ni-Ni_ (~3.07 Å) suggested that the structure of the Ni(II) adsorbed on the CA soil was basically the same as that of Ni(OH)_2_(s), while the Ni-Al shell (*R*_Ni-Al_ ~3.16 Å) gradually formed and grew with the increasing contact time. Under weakly acidic conditions, the sorption mechanism of Ni(II) on the CA soil possibly included at least two processes: (i) a fast accumulation dominated by ion exchange and surface complexation and (ii) the formation of a Ni-Al LDH phase over the long term. A high temperature is beneficial to the fixation of Ni(II) on the CA soil and the formation of a Ni-Al LDH.

The sorption reaction at the solid/liquid interface greatly affects the mobility, speciation, fate and bioavailability of heavy metals in aquatic and soil environments. To understand how these processes are affected by environmental factors and to make sound decisions about environmental remediation and protection, it is imperative to understand the sorption mechanism of heavy metals at solid/liquid interfaces^1^,^2^. Recently, the sorption mechanisms of Ni(II) on clay minerals, oxides, and nanomaterials have been extensively studied using macroscopic and spectroscopic techniques. The main mechanisms that have been postulated for Ni(II) sorption at the solid/water subsurface include ion exchange, surface complexation including inner-sphere complexes (ISCs) and outer-sphere complexes (OSCs), surface-induced precipitation, co-precipitation, and diffusion in particle micropores^3^,^4^,^5^,^6^,^7^,^8^,^9^. Although macroscopic approaches are important tools to understanding the geochemical behaviours of Ni(II) in the environment, spectroscopic approaches such as X-ray photoelectron spectroscopy (XPS) and especially extended X-ray absorption fine structure (EXAFS) are powerful tools whose use is necessary to elucidate the sorption processes of Ni(II) at the molecular scale^2^,^10^,^11^,^12^,^13^,^14^.

As is well known, the sorption mechanism of Ni(II) at the solid/water subsurface is strongly dependent on the contact time, pH, ionic strength, organic matter, Ni(II) concentration in the solid and aqueous phases, and temperature as well as the type of adsorbent^1^,^2^,^5^,^6^,^7^,^8^,^9^,^10^,^15^,^16^,^17^. Elzinga and Sparks^16^ successfully distinguished the different sorption mechanisms of Ni(II) on montmorillonite and pyrophyllite and effectively estimated the distribution of Ni(II) between the minerals in 1:1 and 2:1 mixtures. The results showed that the surface precipitation on the pyrophyllite phase was a more effective mechanism, whilst surface complexation including the formation of OSCs and ISCs was predominant on the montmorillonite surface. However, it is interesting that the sorption of Ni(II) on boehmite mainly formed inner-sphere bidentate mononuclear complexes rather than the expected Ni-Al layered double hydroxide (LDH) phase^9^. The presence of soil fulvic acid (SFA) can clearly enhance the sorption of Ni(II) on boehmite through forming both ligand-bridging ternary surface complexes (i.e., Ni-SFA-boehmite) and metal-bridging ternary surface complexes (SFA-Ni-boehmite)^9^. A mixed Ni-Al hydroxide phase can form on a pyrophyllite surface even at a low surface loading and undersaturated conditions with respect to the formation of the Ni(OH)_2_ (s) phase. At the molecular scale, an EXAFS analysis also showed that the number of Ni second-neighbour atoms at a bond distance of ~3.0 Å increased from 1 to 5 with the increasing Ni surface loading on the pyrophyllite^6^. The results suggested that the pyrophyllite surface could promote hydrolysis and multinuclear complex formation and that the total coverage of the surface was not necessary for the formation of multinuclear surface complexes. The formation kinetics of the Ni-Al LDH phase on a soil clay fraction (Typic-Hapludult) was also successfully explored by the EXAFS approach^1^. The results showed that the Ni-Al LDH precipitate can form within 15 min at pH 7.5, whereas no LDH phase was observed within 72 h at pH 6.0, which clearly indicates the strong pH dependence of the Ni(II) sorption on the clay fraction^1^. Voegelin and Kretzschmar^15^ found that the LDH phases in the Zn(II) and Zn-Ni experiments could be almost completely dissolved in a 0.01 mol/L CaCl_2_ solution at pH ~3.0, whereas the LDH phase was still present in the Ni(II) experiment, in good agreement with the higher resistance of Ni-LDH against dissolution even under acidic conditions, which is very important to evaluating the bioavailability and toxicity of heavy metals in the environment.

Although previous studies have established that metal hydroxide precipitates may form on clay minerals and oxides under specific reaction conditions, it is very difficult to directly extrapolate these results to environmental media. Investigations on the sorption mechanism of Ni(II) on calcareous aridisol (CA) soil, one dominant soil type in northwestern China, are still scarce, yet they are very important to evaluating the geochemical behaviours of Ni(II) in the arid and semi-arid regions of China. Moreover, soil systems are rarely, if ever, at equilibrium, which makes it important to understand the sorption mechanism of Ni(II) over a broad range of contact times, pH values, and temperatures. The objective of the present work is to investigate the retention of Ni(II) on the CA soil in batch experiments, testing the influences of the contact time, pH and temperature and using EXAFS and XPS to assess the retention mechanisms at the molecular level.

## Results

### Sorption investigation

**Figure 1:**
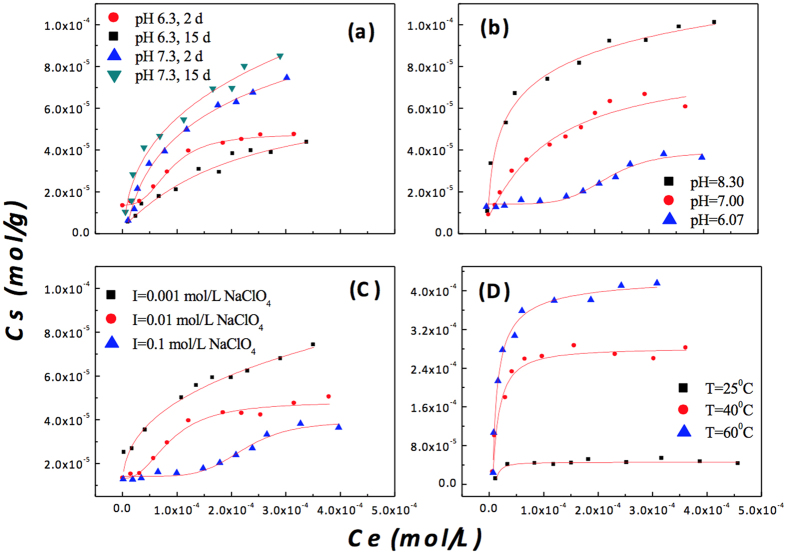
Effects of contact time, pH, ionic strength and temperature on Ni(II) sorption on the CA soil. (**a**) Effect of contact time at different pH. *m/V* = 1.2 g/L, *I* = 0.01 mol/L NaClO_4_, *T* = 25 °C; (**b**) Effect of pH. *m/V* = 1.2 g/L, *I* = 0.01 mol/L NaClO_4_, *T* = 25 °C; (**c**) Effect of ionic strength. *m/V* = 1.2 g/L, *pH* = 6.3 ± 0.1, *T* = 25 °C; (**d**) Effect of temperature. *m/V* = 0.4 g/L, *I* = 0.1 mol/L NaClO_4_, *pH* = 8.2 ± 0.1.

Figure 1a shows that the sorption of Ni(II) on the CA soil increased as the contact time increased from 2 to 15 days at pH ~7.3; however, it slightly decreased at pH ~6.3. Figure 1b shows that the sorption of Ni(II) on the CA soil increased to a large extent and that the shape of the sorption isotherm of Ni(II) somehow changed with the increase in pH value. Moreover, the sorption of Ni(II) on the CA soil decreased with the increasing ionic strength (Fig. 1c). Figure 1d clearly shows that the sorption of Ni(II) on the CA soil increased significantly with the rise in temperature. It is interesting that the enthalpy (∆*H*°) decreased as the initial concentration of Ni(II) increased when *c*[Ni(II)]_initial_ was less than 2.7 × 10^−4^ mol/L (see Table S2 in the supplementary information (SI)). However, ∆*H*° increased slightly again when *c*[Ni(II)]_initial_ was over 2.71 × 10^−4^ mol/L in aqueous solution. The standard Gibbs free energy (∆*G*°) of the sorption reaction became more negative, suggesting that the sorption of Ni(II) on CA soil was favoured under high temperature.

### EXAFS and XPS analyses

EXAFS and XPS approaches were applied to explore the sorption mechanism of Ni(II) on the CA soil surface at the molecular level. The radial structure functions (RSFs) and *k*^*3*^*χ(k*) oscillations of reference samples of Ni(II)_aq_, Ni(OH)_2_(s), Ni-Al LDH and NiO are shown in Fig. S4. The peaks located in the *R* ranges of 1.1 - 2.1 Å and 2.2 - 3.2 Å were assigned to Ni-O and Ni-Ni shells, respectively. The RSFs and *k*^*3*^*χ(k*) oscillations of Ni(II)_aq_ (uncorrected phase shift) clearly deviate from those of Ni(OH)_2_(s), Ni-Al LDH and NiO. The intensity ratio of the fist peak to the second peak clearly decreased in the following order: Ni(II)_aq_ > Ni-Al LDH > Ni(OH)_2_(s) > NiO. This was due to the strong backscatter from the Ni atoms in the crystal structures of Ni-Al LDH, Ni(OH)_2_(s) and NiO.

The *k*^*3*^*χ(k*) functions of the reference samples also revealed that the smoothly decreasing oscillatory patterns of the *k*^*3*^*χ(k*) function were quite different (Fig. S4b). The small beat near 5.0 Å^−1^ and the split at approximately 8.0 Å^−1^ were also observed for both Ni-Al LDH and Ni(OH)_2_(s), which could be attributed to the overlap of the Ni-Ni and Ni-O waves (Fig. S5). Therefore, the small beat at approximately 5.0 Å^−1^ and the split at approximately 8.0 Å^−1^ can be characteristics of LDH and/or Ni(OH)_2_(s) formations. The first peak can be fitted with the Ni-O backscatter pair in the RSFs, whereas Ni-Ni and/or Ni-Al pairs should be taken into account for the second peak. The coordination number (*CN*), interatomic distance (*R*), energy shift (∆*E*_0_), residual factors (*R*_*f*_), and Debye-Waller factors (*σ*^2^) are summarized in the Table 1. The spectrum of Ni(II)_aq_ can be simulated reasonably with a small *R*_*f*_ (1.3%) by assuming a number of *CN*_Ni-O_ ~6.0 at *R*_Ni-O_ ~2.04 Å. Good structural models were also obtained for Ni-Al LDH, Ni(OH)_2_(s) and NiO, with a slightly shorter *R*_Ni-O_ (2.03 Å) found for Ni-Al LDH and Ni(OH)_2_(s) and a longer *R*_Ni-O_ (2.05 Å) for NiO^2^,^9^,^15^,^18^,^19^,^20^. It should be noted that the uncertainties were ±0.02 Å for *R* and ±0.5 for *CN* (Table 1), respectively; and then it is impossible to exactly identify the sorption species of Ni(II) on CA soil only basing on the first coordinated shell due to the similar *R* and *CN* of each possible species. Therefore, the sorption species of Ni(II) son CA soil should be distinguished combining the structure information of the high coordinated shells.

**Figure 2:**
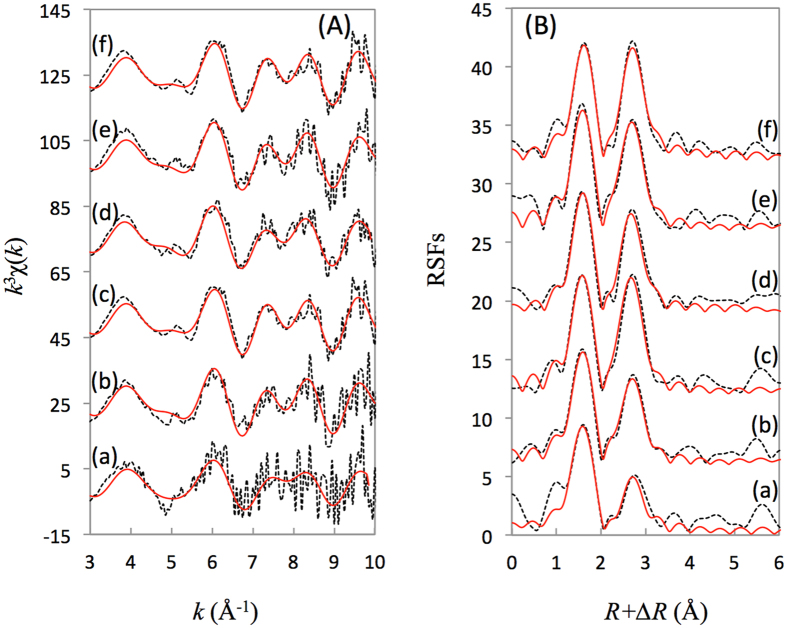
Nickel K-edge EXAFS spectra for Ni(II) adsorbed on the CA soil under different contact times. (**A**) *k*^3^χ(*k*) functions, and (**B**) corresponding RSFs (uncorrected phase shift). (a) pH 6.3, 2 days; (b) pH 6.3, 5 days; (c) pH 6.3, 15 days; (d) pH 7.7, 2 days; (e) pH 7.7, 5 days; and (f) pH 7.7, 15 days. The experimental data (dash line) are fitted (red line) using the parameters described in Table 1.

All the *k*^*3*^*χ(k*) functions had clear beat patterns at approximately 5.0 Å^−1^ except for the sample at pH ~6.3 for 2 days (Fig. 2A). The intensity ratio of the Ni-O shell to the Ni-Ni shell in the RSFs actually decreased to some extent as the contact time increased from 2 to 15 days. At pH ~6.3, the second peak of the Ni adsorbed on the CA soil with a contact time of 2 days can only be fitted well with ~1.5 Si atoms and *R*_Ni-Si_ ~3.20, indicative of typical mono- and/or bi-dentate ISCs. At longer contact times, the second peak tended to be fit well with both Ni-Ni and Ni-Al scattering pairs, which suggests both Ni(OH)_2_(s) and Ni-Al LDH phases. At pH 7.7, the limited dissimilarities for the different contact times suggests that the sorption mechanism of Ni(II) on the CA soil was not significantly changed.

**Figure 3:**
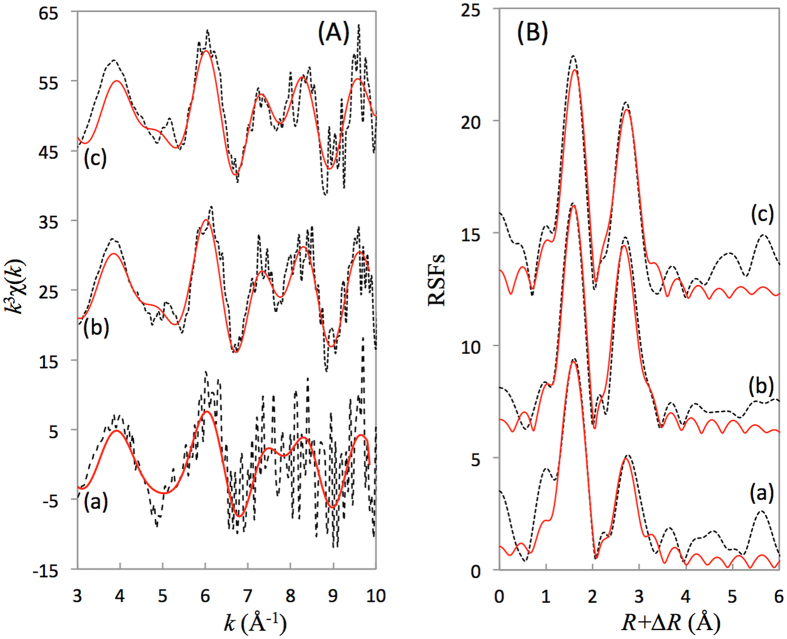
Nickel K-edge EXAFS spectra for Ni(II) adsorbed on the CA soil under different pH values. (**A**) *k*^3^χ(*k*) functions, and (**B**) corresponding RSFs (uncorrected phase shift). (a) pH 6.3, 2 days; (b) pH 7.7, 2 days; (c) pH 10.0, 2 days. Dash line: Experimental data, red line: Fitted data.

Figure 3A shows that a beat pattern at ~5.0 Å is observed above pH ~7.7 in the *k*^*3*^*χ(k*) function, which is the characteristic oscillation of nickel hydroxides including Ni(OH)_2_(s). The second peak was enhanced to some extent with the increase in pH, and the RSFs became more similar to the reference of Ni(OH)_2_(s), which demonstrated that the formation of Ni(OH)_2_(s) will gradually become predominant as the pH increases.

**Figure 4:**
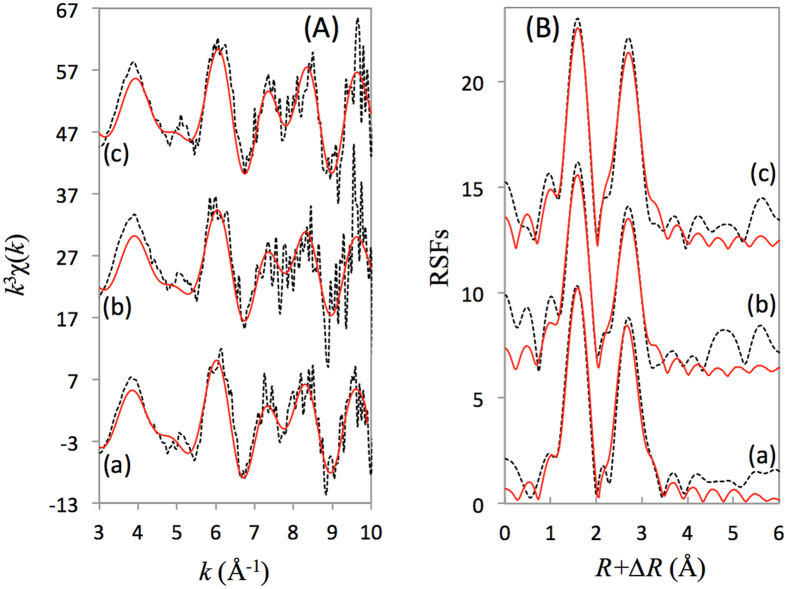
Nickel K-edge EXAFS spectra for Ni(II) adsorbed on the CA soil at pH 7.7 and under different temperatures. (**A**) *k*^3^χ(*k*) functions, and (**B**) corresponding RSFs (uncorrected phase shift). (a) 25 °C; (b) 40 °C; and (c) 60 °C. Dash line: Experimental data, red line: Fitted data.

**Figure 5:**
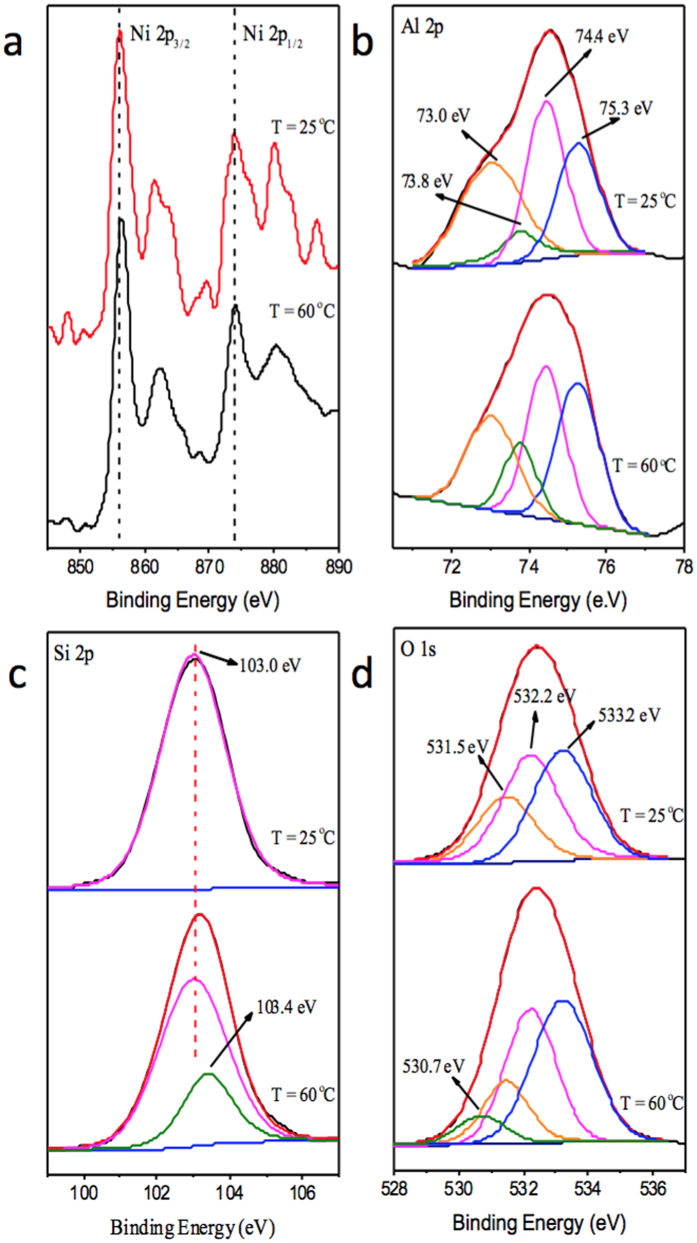
Ni 2p, O 1 s, Al 2p and Si 2p XPS spectra of Ni adsorbed on the CA soil samples at different temperatures. pH = 8.2 ± 0.1. (**a**) Ni 2p, (**b**) Al 2p, (**c**) Si 2p, and (**d**) O 1 s.

EXAFS and XPS approaches were used to study the effect of temperature on the Ni(II) sorption on the CA soil in this presentation (Figs 4 and 5). The *k*^*3*^*χ(k)* functions have a clear beat pattern at 5.0 Å^−1^ (Fig. 4A), which was strengthened as the temperature increased. Moreover, the split near 6.0 Å^−1^ weakened as the temperature increased from 25 to 60 °C. The intensities of the two peaks at 1.53 and 2.67 Å were dependent on the change of temperature in the RSFs (Fig. 4B). The results indicated that the sorption species of Ni(II) on the CA soil was dominantly controlled by precipitation at pH 7.7; however, the formation of the precipitate might change as the temperature rises.

Additional information is provided by the XPS results (Fig. 5). There were double characteristic peaks at 856.1 eV (assigned to 2p_3/2_) and 873.8 eV (assigned to 2p_1/2_) at 25 °C, and the former shifted to a higher binding energy by approximately 0.2 eV as the temperature increased to 60 °C. The shape of Al 2p changed a lot as the temperature increased, and the deconvolution analysis showed that the species with a binding energy at 73.8 eV was increased at high temperature (Fig. 5b), which indicated that more Al atoms possibly participated in the sorption of Ni(II) on the CA soil at high temperature. A similar phenomenon was found in the Si 2p, where the binding energy was increased by ~0.2 eV and a new species may have been formed at high temperature.

## Discussion

Several main mechanisms have been postulated for the slow continued metal sorption by clays and oxides, including the adsorption of heavy metals on solid surfaces that have relatively large activation energies, diffusion into the micropores of the minerals, and the continued growth of a surface precipitate away from the adsorbent surface^5^,^21^,^22^. In the experimental condition, the speciation of Ni(II) in aqueous solution was predominately as Ni^2+^ below pH ~8.0, and the hydrolysis products such as Ni(OH)^+^, Ni(OH)_2_^0^, Ni(OH)_3_^−^, Ni(OH)_4_^2−^, Ni_2_(OH)^3+^, and Ni_4_(OH)_4_^4−^ could be negligible at pH ~7.5 and c[Ni(II)]_initial_ = 1.71 mmol/L^7^. According to the precipitation constant of Ni(OH)_2_(s) (*K*_*sp*_ = 2.0 × 10^−15^), Ni(OH)_2_(s) precipitation can only occur above pH ~7.8^2^. In the sorption system of the current study, the initial concentration of Ni(II) in aqueous phase was much less than 1.71 mmol/L; therefore, the Ni(II) sorption on the CA soil should be dominated by ion exchange and/or surface complexation at acidic conditions; however, in the high pH range, precipitates (i.e., Ni(OH)_2_(s) and Ni-Al LDH) will play very important roles in the fixation of Ni(II) on the CA soil.

A decreasing sorption of Ni(II) under a longer contact time was observed at pH 6.3 (Fig. 1a). This indicated that the fast accumulation of Ni(II) on the CA soil was possibly followed by a partial desorption at pH ~6.3, which resulted in the lower sorption of Ni(II) on CA as the contact time increased^23^. Under acidic conditions, adsorption was the most likely mechanism responsible for the initial fast stage, and then the more stable surface-induced precipitation gradually formed as the contact time increased in the subsequent slower process^16^. Adsorption and precipitation should be considered to the consecutive Ni(II) sorption mechanisms. This could easily be proven from the changes in the RSFs and *k*^*3*^*χ(k*) function under different contact times (Fig. 2 and Table 1). EXAFS analysis suggested that the local atomic structure of Ni(II) on the CA soil surface at low pH was homologous with Ni(II)_aq_ as the ISCs in the initial stage and then increasingly transformed to the neo-formation of Ni(OH)_2_(s) and/or Ni-Al LDH from 5 to 15 days. The Ni(II) sorption on the CA soil at pH ~6.3 thus occurred in at least two visible stages: (i) the fast accumulation of Ni(II) on the CA soil surface through ion exchange and surface complexation, with part of the Ni atoms possibly simultaneously diffusing into the inter-layer and lattice of the CA soil and then providing more unoccupied sorption sites; (ii) then, the surface induced the precipitation of a mixture of Ni-Al LDH and Ni(OH)_2_(s) as the contact time increased.

Under weakly alkaline conditions (pH ~7.3), a slow continued Ni(II) sorption was observed to a lesser extent on the CA soil, which was characteristic of heavy metal sorption on clay and oxides^5^. Meanwhile, EXAFS clearly demonstrated that a precipitate phase of Ni(II) indeed formed at the surface of the CA soil under alkaline conditions. Moreover, the *CN*_Ni-Ni_ decreasing from 6.1 to 4.6 and the *CN*_Ni-Al_ increasing from 2.4 to 4.9 with the contact time increasing from 2 to 15 days (Table 1) indicated that more than two types of precipitates formed at the surface of the CA soil over the long term^2^,^13^,^24^,^25^. As the contact time increased, Ni atoms were gradually and partly substituted by Al to form the more stable phase of Ni-Al LDH, resulting in a clear destructive interference between the Ni and Al backscatter contributions and causing an amplitude cancellation of the Ni and Al shells^25^,^26^. In accordance with the results of Manceau^26^, Al substituting for Ni dampens the second-shell amplitude. Consequently, the fitted coordination numbers below the crystallographic value of 6.0 for the second shell may indicate the presence of Al, whereas a value above 6.0 may indicate the presence of Si. Therefore, the surface-induced precipitates of Ni(OH)_2_(s) and Ni-AL LDH were mainly responsible for the continuous sorption of Ni(II) on the CA soil in alkaline conditions. The driving forces for the continuous growth of surface precipitate should include the presence of Ni(II) in solution and the increasing surface area due to the formation of precipitates. As the contact time increased from 1 to 7 days, the amount of Ni(II) on the surface increased and formed second coordination shells in the RSFs^27^. The sorption of Ni(II) on the pyrophyllite surface may also have experienced two different stages: (i) the initial formation of the Ni-Al LDH phase, where Al was derived from the pyrophyllite structure, and (ii) the ultimate formation of a Ni-Al phyllosilicate phase due to the incorporation of Si (derived from the pyrophyllite) into the Ni-Al LDH interlayer^6^,^17^. The latter process could be viewed as a silication of the LDH structure. The formation of a Ni-Al LDH phase was a necessary intermediate step towards the formation of a more stable phyllosilicate-type phase in Al-bearing clay mineral systems^28^,^29^.

Figures 1b and 3 showed a clear effect of the pH on the Ni(II) sorption on the CA soil, and the sorption mechanism of Ni(II) was quite complicated, including ion exchange, surface complexation, and precipitation. EXAFS analysis identified that surface-induced precipitates of the Ni(OH)_2_(s) and Al-Ni LDH phases were predominant at high ionic strength (Fig. S7). Previous studies have proven that small clusters of nickel hydroxide with a high percentage of octahedra at edge positions and therefore with fewer than six neighbours (i.e., O atoms) or the presence of additional OSCs of Ni(II) without neighbouring backscatter atoms might reduce the statistical coordination number as well^22^. These features confirmed the presence of more than one ordered neighbour shell around each Ni atom. However, the mono- and/or bi-dentate ISCs were the predominant species for Ni(II) sorption on CA soil at pH ~6.3 at 2 days. The formation of more stable phases of Ni(OH)_2_(s) and Ni-Al LDH became more important for Ni(II) sorption when the contact time increased above 5 days.

The unusual change in ∆*H*° suggested a different sorption mechanism for Ni(II) on the CA soil as the initial concentration of Ni(II) increased (Table S2). Surface complexation and/or ion exchange may have contributed to Ni(II) sorption on the CA soil in a small range of Ni(II) concentration; however, the surface-induced precipitates of Ni(OH)_2_(s) and Ni-Al LDH gradually formed and caused a large ∆*H*° over a larger range of Ni(II) concentration. As the temperature increased, the more negative ∆*G*° confirmed that more stable species of Ni(II) were possibly formed at high temperature, for example, Ni-Al LDH phases^17^.

At different temperatures, EXAFS analysis showed a high similarity in the *R*_Ni-O_ ~2.03 Å, even at 60 °C (Table 1), which suggested that precipitation should be the main sorption mechanism. Based on the structure parameters deduced from EXAFS (Table 1), the environment of the Ni(II) adsorbed on the CA soil at 25 °C was basically similar to Ni(OH)_2_(s), whereas a mixture local atomic structures of Ni(OH)_2_(s) and Ni-Al LDH gradually formed due with the *CN*_Ni-Al_ increasing from 1.6 to 2.9 as the temperature increased from 25 to 60 °C^2^,^13^,^24^,^25^,^26^. Another possibility is that the dissolution of minerals in the CA soil was enhanced at high temperature, leading to an increase of the availability of Al^3+^ in the aqueous phase, which was beneficial to the formation of Ni-Al LDH.

The Ni 2p XPS spectra were influenced exclusively by the charge transfer process from the nearest-neighbour atoms^30^. The shift of the binding energy of Ni 2p_3/2_ (Fig. 5a) further confirmed the change of the formation of the Ni(II)-adsorbed CA soil surface associated with the increase in temperature. The shape and the binding energy of the Al 2p spectrum were different between 25 °C and 60 °C, and the new speciation at 73.8 eV at 60 °C was possibly related to the formation of Ni-Al LDH (Fig. 5b). Moreover, the change in Si 2p (Fig. 5c) suggested that Si possibly participated in the sorption of Ni(II) on the CA soil to form Ni-Si phyllosilicate; however, EXAFS analysis could not effectively distinguish the contribution of the Ni-Si pair to the second peak in the RSFs from that of the Ni-Al pair. It suggested that the contribution of Ni-Si phyllosilicate was quite limit to the sorption of Ni(II) on the CA soil under the experimental conditions. Wang *et al*.^31^ found that the interaction between Ni(II) and Na-mordenite was mainly dominated by ion exchange at low temperature, whereas other interactions (such as precipitation and/or co-precipitation) might lead to a continuous sorption of Ni(II) at high temperature. Ni(II) sorption on CA soil exhibited a positive response to the increasing temperature.

In this study, batch experiments and EXAFS analysis showed that the sorption mechanism of Ni(II) on the CA soil was strongly dependent on the pH, contact time, and temperature. The formation of ISCs and/or ion exchange might be mainly responsible for Ni(II) sorption on the CA soil in the short term, and the formation of Ni-Al LDH and Ni(OH)_2_(s) gradually increased to some extent with the increase in contact time. As the pH increased, a Ni(OH)_2_(s) phase formed, which can transform into a more stable phase of Ni-Al LDH in the long term. When the temperature was increased in the experimental system, a greater contribution from Ni-Al LDH to the retention of Ni(II) on the CA soil will be observed due to the high availability of Al^3+^ and more stable formation of Ni-Al LDH in comparison with Ni(OH)_2_(s). These findings are critical to understanding the geochemical behaviours of Ni(II) in arid and semi-arid region soils, which are suffering from serious pollution in China.

## Methods

### Materials

All chemicals used in the experiments were purchased in analytical purity and used without any further purification. CA soil (0–20 cm in depth) was collected from Yumen County (Gansu province, China). CA soil was sieved and through 300-mesh, and then washed with Milli-Q water (18.2 MΩ·cm) in order to get the fine particles before the sorption and characterization experiments. Mineral compositions were 11% chlorite, 19% hydromica, 31% gypsum, 22% quartz, 13% feldspar, and 4% calcite. The N_2_-BET surface area of the CA soil was measured to be 6.17 m^2^/g. CA soil has a large CEC (cation exchange capacity) of 90.7 meq/100 g (Table S1), indicating that ion exchange may be an important mechanism for Ni(II) sorption in low pH range. The content of organic matters is about 0.87%, and the pH of CA soil is about 7.97. The other characterizations were discussed widely in the SI.

### Batch sorption

Stock suspension of CA soil was shaken with 0.1, 0.01, and 0.001 mol/L NaClO_4_ solution for 24 hours in the 10.0 mL polyethylene tubes to pre-equilibrate with Na^+^. Then, different volumes of Ni(II) stock solution were spiked into the above test polyethylene tubes to maintain the initial concentration of Ni(II) over the range from 1.6 × 10^−5^ mol/L to 6.0 × 10^−4^ mol/L, and the final ratio of solid-to-volume and the total volume of the sorption systems were setup at 1.2 g/L (or 0.4 g/L for the effect of temperature) and 6.0 mL, respectively. The expectant pH (pH~6.0, 7.0 and 10.0) was adjusted by adding negligible volume of 0.1 or 0.01 mol/L HClO_4_ and NaOH solutions. After the above sorption systems were shaken for 2 or 15 days at a given temperature, the solid and liquid phases were separated by centrifugation at 14,000 rpm for 30 minutes. The solid were collected for XPS analysis. Ni(II) concentration was analyzed using Perkin-Elmer Analyst 300 atomic absorption spectrophotometer equipped with air acetylene burner. The analytical wavelength was set at 232 nm and hollow cathode lamp was operated at 20 mA.

### EXAFS Analysis

The procedures of Ni(II) adsorbed samples are described in the SI. The reference sample of Ni(II)_aq_ was obtained from dissolving higher purity nickel powder using nitric acid. Ni(OH)_2_(s) and Ni-Al LDH were synthesized according to the references^27^,^32^. A series set of Ni K-edge X-ray absorption spectra at 8333 eV were recorded at the Shanghai Synchrotron Radiation Facility (SSRF, Shanghai, China). The ionization chambers with N_2_ or Ar atmosphere were used to collect the Ni K-edge spectra in fluorescence mode at ambient temperature. The normalization of X-ray absorption spectra, extraction of EXAFS oscillations and data analysis were performed following standard procedures by using ATHENA and ARTERMIS interfaces to the IFFEFIT software^33^,^34^. The resulting χ(*k*) functions were weighted with *k*^*3*^ to compensate for the dampening of the EXAFS amplitude with the increasing *k* and were Fourier transformed to obtain the RSFs. The amplitude reduction factor, *(S*_*0*_^2^), was fixed at 0.85. A good fit was determined on the basis of the minimum residual error (*R*_*f*_). The theoretical backscatter phases and amplitudes used in data analysis were calculated with the scattering code FEFF 7.0 using the crystal structures of Ni(OH)_2_^35^, NiO^36^ and NiAl_2_O_4_^37^.

## Additional Information

**How to cite this article:** Qiang, S. *et al*. Sorption of Nickel(II) on a Calcareous Aridisol Soil, China: Batch, XPS, and EXAFS Spectroscopic Investigations. *Sci. Rep.* **7**, 46744; doi: 10.1038/srep46744 (2017).

**Publisher's note:** Springer Nature remains neutral with regard to jurisdictional claims in published maps and institutional affiliations.

## Supplementary Material

Supplementary Information

## Figures and Tables

**Table 1:** Best-Fit Structural Parameters Derived from EXAFS Analysis of Ni(II) Adsorbed CA Soil Samples and Reference Samples^*^.

Samples	Shell	CN	R (Å)	σ^2^ (Å^2^)	∆E_0_ (eV)	R_f_ (%)
Ni(II)_aq_	Ni-O	6.0	2.04	0.007	−0.2	1.3
Ni(OH)_2_(s)	Ni-O	6.0	2.03	0.007	−1.1	1.1
	Ni-Ni	6.0	3.09	0.008		
NiO	Ni-O	6.0	2.05	0.007	−2.0	1.3
	Ni-Ni	12.0	2.92	0.008		
Ni-Al LDH (Ni/Al = 3:1)	Ni-O	6.0	2.03	0.008	2.8	4.9
	Ni-Ni	3.5	3.06	0.005		
	Ni-Al	2.5	3.12	0.003		
(1) 5 d, pH7.7	Ni-O	6.1	2.03	0.007	−1.0	1.5
	Ni-Ni	6.5	3.06	0.006		
	Ni-Al	4.6	3.16	0.006		
(2) 15 d, pH7.7	Ni-O	5.8	2.05	0.007	0.7	1.2
	Ni-Ni	4.9	3.07	0.006		
	Ni-Al	1.2	3.18	0.006		
(3) 5 d, pH6.3	Ni-O	5.9	2.03	0.007	−0.8	0.7
	Ni-Ni	5.2	3.06	0.007		
	Ni-Al	2.5	3.14	0.008		
(4) 15d, pH6.3	Ni-O	6.1	2.03	0.007	−0.9	0.3
	Ni-Ni	5.1	3.05	0.007		
	Ni-Al	2.9	3.27	0.007		
(5) pH 6.3, 2days	Ni-O	5.7	2.04	0.007	3.1	4.2
	Ni-Si	1.5	3.20	0.004		
(6) pH 7.7, 2days	Ni-O	5.5	2.04	0.006	−1.0	3.1
	Ni-Ni	6.1	3.07	0.007		
	Ni-Al	1.6	3.13	0.007		
(7) pH 10.0, 2days	Ni-O	6.2	2.04	0.007	0.8	2.3
	Ni-Ni	5.4	3.08	0.007		
	Ni-Al	1.1	3.12	0.007		
(8) 40 °C, pH 7.7, 2 d	Ni-O	5.9	2.03	0.008	−1.3	1.1
	Ni-Ni	4.8	3.06	0.007		
	Ni-Al	1.6	3.16	0.007		
(9) 60 °C, pH 7.7, 2 d	Ni-O	6.2	2.03	0.007	−0.5	0.9
	Ni-Ni	4.9	3.06	0.006		
	Ni-Al	2.9	3.16	0.006		

*R: Interatomic distance, *CN*: Coordination number, *σ*^*2*^: Debye-Waller factor, ∆*E*_*0*_: Energy shift, *R*_*f*_: The residual factor *R*_*f*_ = 100 × ∑_*k*_(*k*^3^*x*_exp_-*k*^3^*x*_calc_)/∑_*k*_(*k*^3^*x*_cal_) measures the quality of the model Fourier-filtered contribution (*x*_calc_) with respect to the experimental contribution (*x*_exp_). Errors in the fitted parameters were estimated to be generally ±0.02 Å for *R* and ±0.5 for *CN*.
