# Supplementary material for: Sorption of Nickel(II) on a Calcareous Aridisol Soil, China: Batch, XPS, and EXAFS Spectroscopic Investigations
Source: Sci Rep. 2017 Apr 25;7:46744. doi: 10.1038/srep46744 (PMC5404261; doi:10.1038/srep46744)
Supplement: Supplementary Information [file srep46744-s1.pdf]

Supplementary Information on

Sorption of Nickel(II) on a Calcareous Aridisol Soil, China: Batch, XPS, and EXAFS Spectroscopic Investigations

Shirong Qiang[1,2], Bin Han[1,4], Xiaolan Zhao[1,4], Yunbo Yang[1,4], Dadong Shao[3], Ping Li[1], Jianjun Liang[1], Qiaohui Fan[1,*]

[1]Key Laboratory of Petroleum Resources, Gansu Province / CAS Key Laboratory of Petroleum Resources Research, Institute of Geology and Geophysics, Chinese Academy of Sciences, Lanzhou, Gansu, 730000, China; [2]Key Laboratory of Preclinical Study for New Drugs of Gansu Province, and Institute of Physiology, School of Basic Medical Sciences, Lanzhou University, 199 Donggang West Road, Lanzhou 73000, China; [3]Institute of Plasma Physics, Chinese Academy of Sciences, Hefei 230031, China; [4]Graduate University of Chinese Academy of Sciences, Beijing, 100049, China.

* Corresponding author. Tel: +86-931-4960831; E-Mail: fanqh@lzb.ac.cn or fanqiaohui@gmail.com;

Shirong Qiang: qiangshirong@lzu.edu.cn
Bin Han: 379176163@qq.com
Xiaolan Zhao: zhao_xl08@163.com
Yunbo Yang: 1157565244@qq.com
Dadong Shao: shaodadong@126.com
Ping Li: lipingls06@126.com
Jianjun Liang: liangjj@lzb.ac.cn
Qiaohui Fan: fanqiaohui@gmail.com

SI 1. XRD, FT-IR, SEM, TEM and XPS analysis

Calcareous aridisol (CA) soil was characterized by Fourier Transform Infrared (FT-IR) spectroscopy, X-ray diffraction (XRD), scan electron microscopy (SEM), and transmission electron microscopy (TEM). FT-IR measurement was mounted on a Bruker EQUINOX55 spectrometer (Nexus) in KBr pellet at room temperature. XRD pattern of the CA soil was obtained from the Panalytical X'Pert PRO equipment with a rotation anode using Cu Kα radiation (λ = 0.15406 nm). XRD device was operated at 40 kV and 80 mA, and the measurements were carried out in the range of $2^0 \leq 2\theta \leq 70^0$. SEM images were collected using a Hitachi S-4800 type instrument. Prior to analysis, the CA soil was sprinkled onto adhesive carbon taps supported on metallic disks. TEM images were taken of each sample using a Philips CM10 type microscope at 200 kV. A Rh–Co grid was dipped in a glue solution and representative sample was distributed over the grid. The batch filtered samples were dried at $T = 25 \pm 1$ °C under N_2 conditions before the XPS analysis. The XPS data were obtained with an ESCALab220i-XL electron spectrometer from VG Scientific using 300 W Mg Kα radiation. The pressure in the analysis chamber was maintained below 3×10^{-9} mbar. The binding energies of carbon species, i.e., C 1s line at 284.8 eV, was used to correct the observed binding energies for surface charging.

Fig. S1 showed the typical SEM and TEM images of the CA soil. CA soil has sheet-like amorphous aggregates and a layer structure, which results in the rough surface of the CA soil (Fig. S1a). The special structure of CA soil indicates that it owns strong adsorption ability and high capacity for metal ions. Form Fig. S1b, it is clearer that CA soil exhibits irregular layer structure and platy shape, and the irregular size is about 200-500 nm.

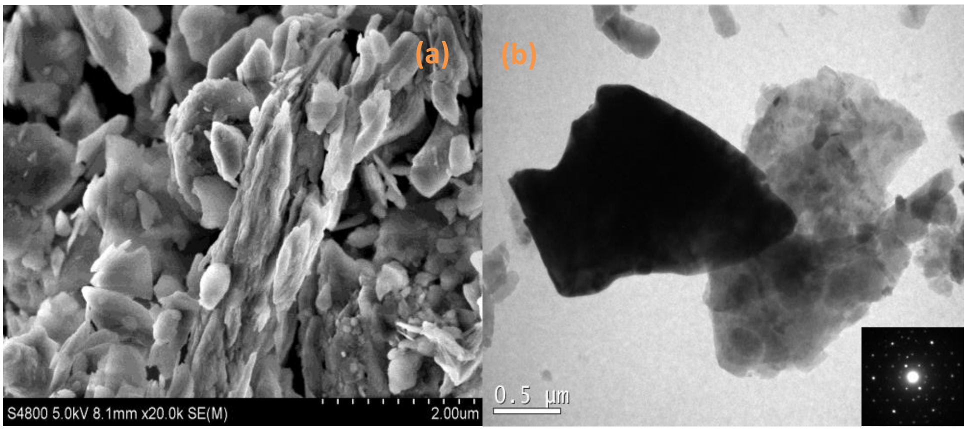

Fig. S1 SEM and TEM images of the CA soil. (a): SEM, and (b): TEM.

The chemical components of the CA soil analyzed by X-ray fluorescence spectroscopy are: SiO_2 50.49%, Al_2O_3 11.19%, Fe_2O_3 4.96%, MgO 6.82%, CaO 10.37%, Na_2O 1.61%, K_2O 2.48%, C 11.64%, S 1.5%, P 0.06% and Ti 0.34%. The mineral compositions are: Chlorite 11%, Hydromica 19%, Gypsum 31%, Quartz 22%, Feldspar 13% and Calcite 4%. Some other chemical and physical properties are listed in Table S1. The main mineral compositions are gypsum, quartz, hydromica, feldspar, chlorite and calcite (Fig. S2). The reflections (2θ = 13.52^0, 19.84^0, 23.42^0, 25.24^0, and 32.22^0) suggest that feldspar is also present in the CA soil. The peaks at 8.88^0, 15.47^0, 17.84^0, 24.31^0 and 27.71^0 correspond to hydromica. The reflections (2θ = 11.7^0, 20.92^0, 31.02^0, 33.34^0, 36.6^0 and 43.36^0) are assigned to the characteristic peaks of gypsum. The intergrowth-minerals of calcite, chlorite and quartz are also found in the sample and are marked in Fig. S2 (1-3).

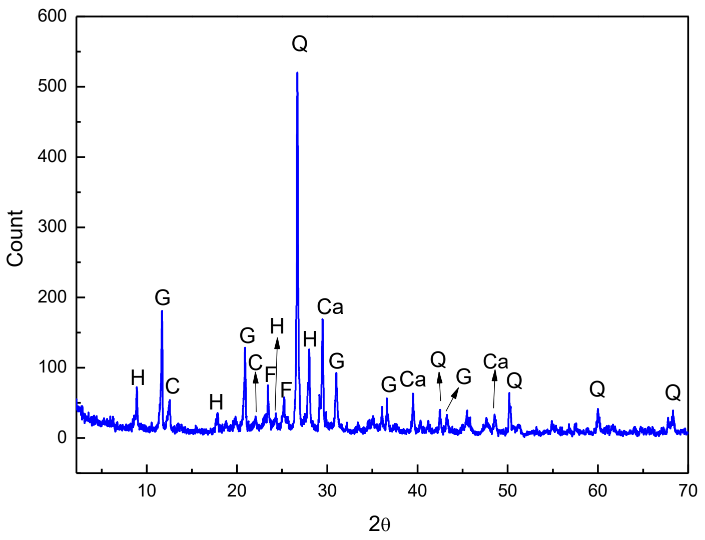

Fig. S2 XRD pattern of the CA soil. C: Chlorite, Ca: Calcite, F: Feldspar, G: Gypsum, H: Hydromica and Q: Quartz.

Table S1. Some chemical and physical properties of the CA soil

OM[a]	Carbonate	pH (1:1)	Conductivity	CEC[b]
0.87%	12%	7.97	4.61mS/cm	90.72meq/100g

a: Organic matter; b: Cation exchange capacity

The FT-IR spectrum of the CA soil is shown in Fig. S3. The peak at 3620 cm^{-1} is assigned to the stretching vibration of Al-O-H. The peaks at 1030 cm^{-1} and 467 cm^{-1} are attributed to Si-O-Si bonds, and the peak at 779 cm^{-1} corresponds to the stretching vibration of δ(O-Si). The peak at 528 cm^{-1} is assigned to O-P-O out-of-plane bend vibrations. The peaks at 3420 cm^{-1} and 1630 cm^{-1} are assigned to the stretching vibration and the bend vibration of water, respectively.

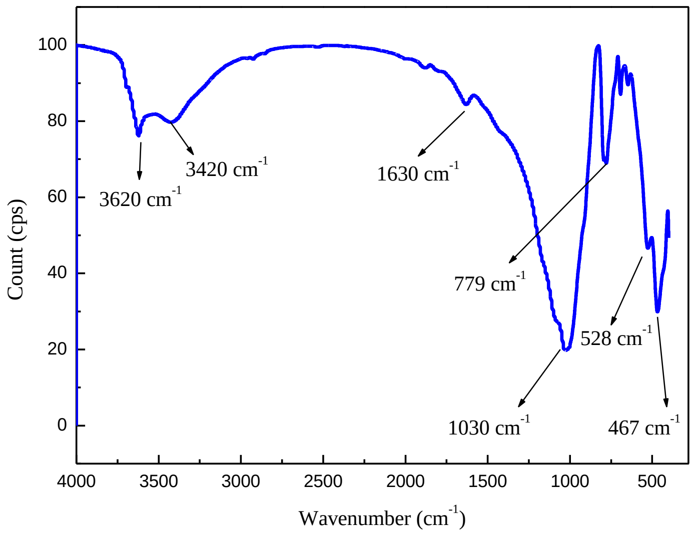

Figure S3 FT-IR analysis of the CA soil.

SI 2. EXAFS samples preparation, data collection and analysis

For EXAFS analysis, NiO powder was purchased from Sinopharm Chemical Regent Co. The standard sample of Ni(II)$_{aq}$ was obtained from dissolving higher purity nickel powder (Sinopharm Chemical Regent Co.) using nitric acid. The $Ni(OH)_2$(s) was prepared by adding 550 mL 30% ammonia to 500 mL of 1.0×10^{-3} mol/L $Ni(NO_3)_2$ solution. The mixture solutions were vigorously stirred and purged N_2; after 2 hours, the suspension was centrifuged and washed with Milli-Q water in six cycles, shock-freezed in liquid N_2 and freeze-dried. Ni-Al LDH sample was prepared by controlled hydrolysis according to reference (4). The amounts of Ni and Al (added as $Ni(NO_3)_2{\cdot}9H_2O$ and $Al(NO_3)_3{\cdot}9H_2O$) were adjusted to give Ni/Al ratio (3:1), then 400 mL of both solutions were combined in a 500 mL flask under vigorous stirring. The pH was gradually raised to 6.9 by discontinuous addition of 2.5 mol/L NaOH and then kept constant for 5 hours using an auto-titrator. The precipitation was washed with Milli-Q water and dried as before. The Ni(II) adsorbed CA soil samples at different conditions were conducted using 500 mL vessels with 10.0 g/L CA soil suspension, 0.01 or 0.1 mol/L $NaClO_4$ and 1.71 mmol/L Ni(II) stock solution, and

added 0.1 and 0.01 mol/L NaOH solution to balance the acidity of Ni(II) solution. The base solution was added firstly, and then the Ni(II) stock solution was introduced as: 10-50 μL increments of the Ni(II) stock solution were introduced into the suspension under constant stirring to disperse the small aliquot of Ni(II) solution. Periods of a few minutes between the increments were chosen to avoid the local concentration of Ni(II) exceeding the solubility limit of $Ni(OH)_2$(s), while allowing the completion of Ni(II) addition in reasonable delay (5). Finally, the suspension was allowed to equilibrate for 2 days. The samples were recovered by filtration using 0.25 μm membrane, and sealed into the polyethylene bag for EXAFS measurement. It was noted that all the metal solutions were purged with N_2 and NaOH solution was freshly prepared to minimize the uptake of carbonate.

A series set of Ni K-edge X-ray absorption spectra at 8333 eV were recorded at the Shanghai Synchrotron Radiation Facility (SSRF, China). The beam line energy of X-ray was equipped with a fixed-exit double-crystal Si (111) monochromator, the electron beam energy in the storage ring was 3.5 GeV, and a maximum current of 220 mA was used. The ionization chambers with N_2 or Ar atmosphere were used to collect the Ni K-edge spectra in fluorescence mode at ambient temperature. A 29-element pixel high purity Ge solid-state detector was used to collect the fluorescence signal. Prior data collection, the energy was calibrated to the first inflection point on the K absorption edge of a Ni foil standard (E_0 = 8333 eV). Solid samples were loaded into individual acrylic holder, whereas a special quartz container was applied to hold the aqueous standards. The data were collected at the Ni K-edge over the energy range of 8233–9322 eV.

Addition and normalization of X-ray absorption spectra, extraction of EXAFS oscillations and data analysis were performed with ATHENA and ATERMIS interfaces to the IFFEFIT software. The EXAFS oscillations were isolated from the raw, averaged data by removal of the pre-edge background, approximated by a first-order polynomial. The energy axis (eV) was converted to photoelectron wave vector units ($Å^{-1}$) by assigning the origin, E_0, to the first inflection point of the absorption edge. The resulting (k) functions were weighted with k^3 to compensate for

the dampening of the EXAFS amplitude with increasing k and were Fourier transformed to obtain radial structure functions (RSFs). The amplitude reduction fact, $(S_0)^2$, was fixed at 0.85. A good fit was determined on the basis of the minimum residual error (R_f). The Debye-Waller factor (σ^2) and energy shift (ΔE_0) were allowed to vary during this optimization. The theoretical backscatter phases and amplitudes used in data analysis were calculated with the scattering code FEFF 7.0 using the crystal structures of $Ni(OH)_2$ (6), NiO (7) and $NiAl_2O_4$ (8).

SI 3. Effect of temperature on Ni(II) sorption on the CA soil

From the thermodynamic data (see Table S2), the values of enthalpy change (ΔH^o) and entropy change (ΔS^o) are calculated from the slope and intercept of the plots of $logK_d$ vs. $1/T$ by the following equations:

$$K_d\ (mL/g) = \frac{equilibrium\ amount\ of\ Ni\ sorbed}{equilibrium\ amount\ of\ Ni\ in\ solution} \times \frac{V}{m} = \frac{C_s}{C_e} \times \frac{V}{m} \qquad (1)$$

$$logK_d = \frac{\Delta S^0}{R} - \frac{\Delta H^0}{RT} \qquad (2)$$

The Gibbs free energy change (ΔG^o) was calculated from the equation:

$$\Delta G^0 = \Delta H^0 - T\Delta S^0 \qquad (3)$$

where V is the solution volume (mL) and m is the solid mass (g); C_s is the amount of Ni(II) adsorbed on per weight unit solid after equilibrium (mol/g); and C_e is the equilibrium concentration of Ni(II) remained in solution (mol/L). R (8.3145 $J \cdot mol^{-1} \cdot K^{-1}$) is the ideal gas constant, and T (K) is the temperature in Kelvin. From Table S2, the positive ΔH^o values mean that the holistic process of Ni(II) from solution to the CA soil is endothermic. The ΔG^o values are negative as expected for a spontaneous process under the conditions applied. ΔG^o becomes more negative with increasing temperature, which indicates that more efficient sorption occurs at higher temperature. At higher temperature, cations are readily desolvated and hence their sorption becomes more favorable (5). The kinetic process of Ni(II) sorption possibly experienced two steps: (1) the hydration sheath of Ni(II) in solution has to be destroyed before its sorption to the surface of CA soil, which requires energy (Q_1); (2)

then the dehydration cations attach to the surface sorption sites on CA soil and release some energy (Q_2). If the energy of dehydration (Q_1) exceeds the exothermicity of cations attaching to the surface (Q_2) (i.e., $Q_1 > Q_2$), the sorption is an endothermic process. It is interesting that ΔH^0 reduces with increasing Ni(II) initial concentrations (at $C[Ni(II)]_{initial} < 2.71\times10^{-4}$ mol/L). It could be qualitatively explained by considering that the sorption occurs initially in the most active available sites involving high interaction energies. At low concentration of Ni(II), sorption is mainly governed by interaction between Ni(II) and the surface of CA soil; a plausible interpretation could be the differences in attractive forces between Ni(II) and sorption sites on the CA soil (9). However, the values of ΔH^o increase slightly with increasing Ni(II) concentration at $C[Ni(II)] > 2.71\times10^{-4}$ mol/L under applied conditions. The possible explanations are: (1) recrystallization to a more stable phase (e.g., $Ni(OH)_2$ and Ni-Al LDH); (2) changes in the elemental composition of a solid-solution (e.g., the Ni/Al ratio of LDH); (3) transformation of LDH to Ni-Al phyllosilicate (10,11).

Table S2. Thermodynamic parameters of Ni(II) sorption on the CA soil. m/V = 0.4 g/L, I = 0.1 mol/L $NaClO_4$, pH = 8.2 ± 0.1

C_0	ΔH^o	ΔS^o	ΔG^o (kJ·mol^{-1})		
(mol/L)	(kJ·mol^{-1})	(J·mol^{-1}·K^{-1})	298.15 K	313.15 K	333.15 K
1.02E-04	36.64	143.99	-6.29062	-9.89037	-11.3303
1.36E-04	33.21	131.93	-6.12493	-9.42318	-10.7425
1.69E-04	31.58	126.22	-6.05249	-9.20799	-10.4702
2.03E-04	30.49	120.98	-5.58019	-8.60469	-9.81449
2.71E-04	26.04	105.83	-5.51321	-8.15896	-9.21726
3.39E-04	27.39	109.09	-5.13518	-7.86243	-8.95333
4.07E-04	28.08	110.56	-4.88346	-7.64746	-8.75306
4.75E-04	30.62	103.61	-0.27132	-2.86157	-3.89767

SI 4. EXAFS analysis of reference samples

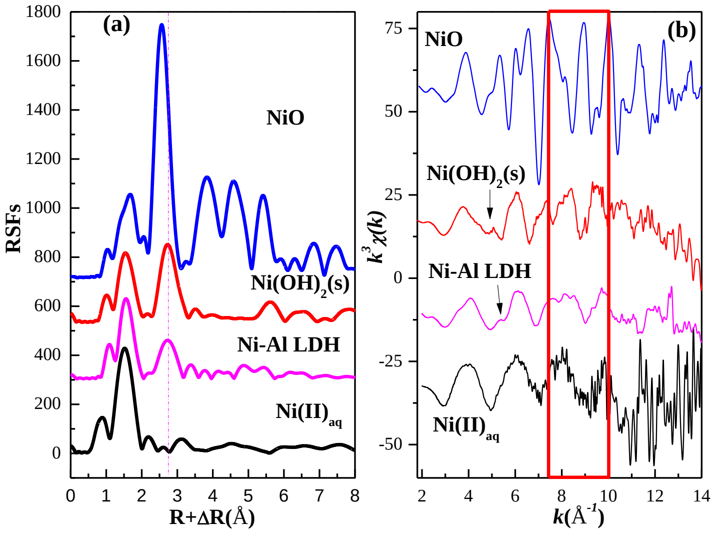

Figure S4 Corresponding Fourier transforms (a) and k^3-weighed Ni K-edge EXAFS for Ni(II) in aqueous solution ($Ni(II)_{aq}$), $Ni(OH)_2(s)$, Ni-Al LDH and NiO samples.

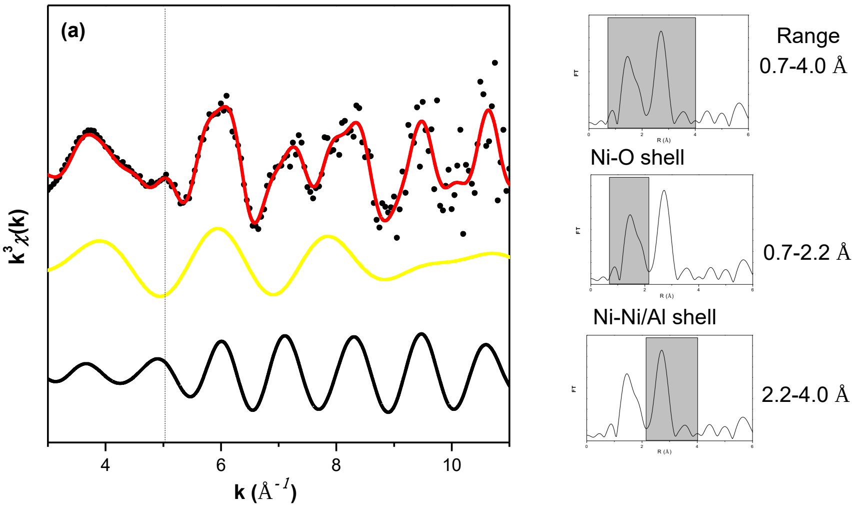

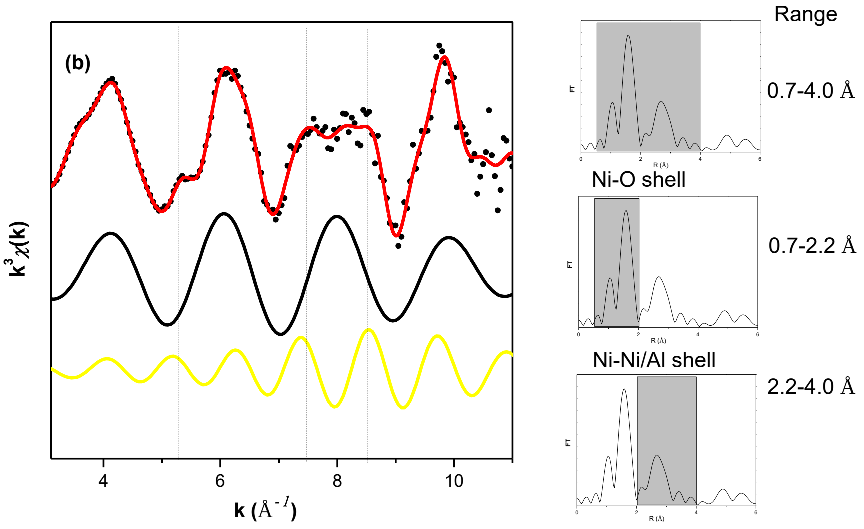

Fig. S5 The upper spectra show the sum of Fourier filtered Ni-O and Ni-Ni contributions (black dot) with theoretical spectrum (red line) for simulation of $Ni(OH)_2$ (a) and Al-Ni LDH (b). Middle and lowest spectra show the Fourier filtered Ni-O and Ni-Ni shell contributions, respectively. The solid lines and scatters are, respectively, model and experimental data.

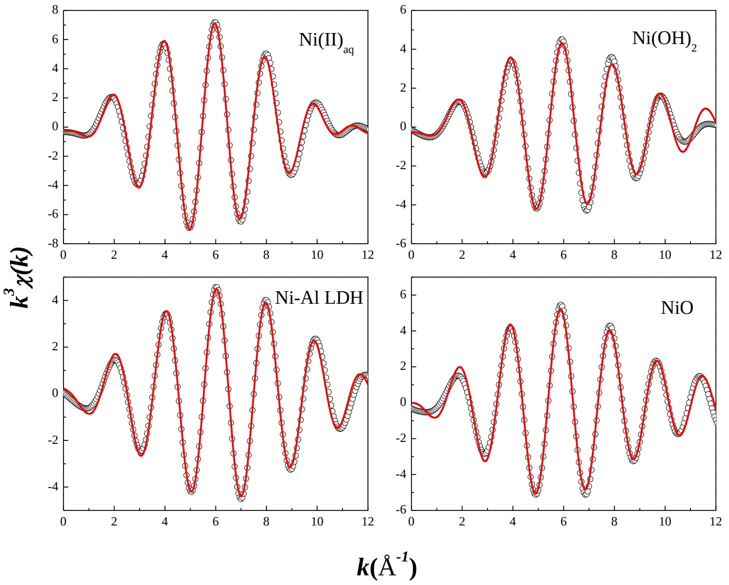

Fig. S6 Experimental (open circles) and model (solid line) Fourier-filtered $k^3\chi(k)$ contribution for the next-nearest backscatter shells at R distance spanning the [0.98, 2.01Å] interval for reference samples.

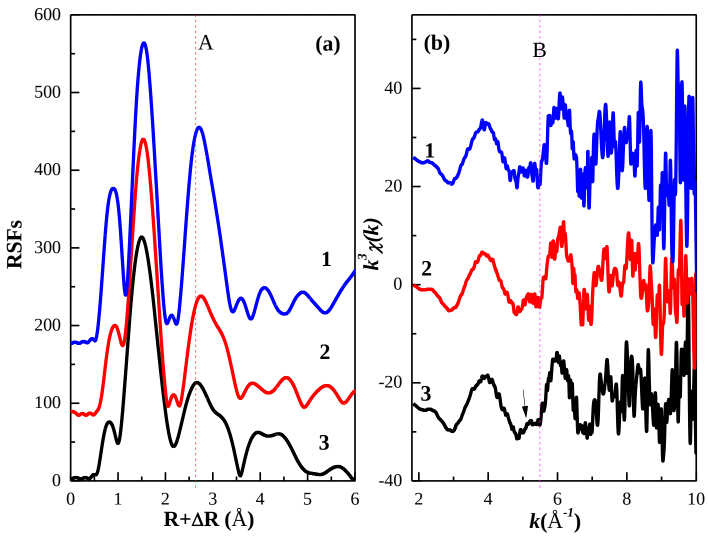

Fig. S7 Corresponding Fourier transforms (a) and k^3-weighed Ni K-edge EXAFS for Ni(II) sorption to CA soil at pH 6.7±0.1 for different ionic strengths. **1**: 0.1mol/L $NaClO_4$; **2**: 0.01mol/L $NaClO_4$, **3**: 0.001 mol/L $NaClO_4$.

References

(1) Sezer, G. A.; Türkmenoğlu, A. G.; Göktürk, E. H. Mineralogical and sorption characteristics of Ankara Clay as a landfill liner. *Appl. Geochem.* **2003**, *18*, 711-717.

(2) Türkmenoğlu, A. G.; Yavuz-Işık, N. Mineralogy, chemistry and potential utilization of clays from coal deposits in the Kütahya province, Western Turkey. *Appl. Clay Sci.* **2008,** *42*, 63-73.

(3) Zachara, J. M.; Smith, S. C.; Liu, C.; McKinley, J. P.; Serne, R. J.; Gassman, P. L. Sorption of Cs^+ to micaceous subsurface sediments from the Hanford site, USA. *Geochim. Cosmochim. Acta* **2002**, *66*, 193-211.

(4) Scheinost, A. C.; Sparks, D. L. Formation of layered single- and double-metal hydroxide precipitates at the mineral/water interface: A multiple-scattering XAFS analysis. *J. Colloid Interf. Sci.* **2000**, *223*, 167-178.

(5) Fan, Q. H.; Tan, X. L.; Li, J. X.; Wang, X. K.; Wu, W. S.; Montavon, G. Sorption of Eu(III) on attapulgite studied by batch, XPS, and EXAFS techniques. *Environ. Sci. Technol.* **2009**, *43*, 5776-5782.

(6) Natta, G. Constitution of hydroxides and hydrates. *Gazzetta Chimica Italiana*. **1928**, *58*, 344-358.

(7) Sasaki, S.; Fujino, K.; Takéuchi, Y. X-ray determination of electro-density distributions in oxides, MgO, MO, CoO, and NiO, and atomic scattering factors of their constituent atoms *Proc. Japan Acad.* **1979**, *55*, 43-48.

(8) Roelofsen, J. N.; Peterson, R. C.; Raudsepp, M. Structural variation in nickel aluminate spinel ($NiAl_2O_4$). *Am. Min.* **1992**, *77*, 522-528.

(9) Mihoubi, D.; Bellagi, A. Thermodynamic analysis of sorption isotherms of bentonite. *J. Chem. Thermodyn.* **2006**, *38*, 1105-1110.

(10) Scheinost, A. C.; Ford, R. G.; Sparks, D. L. The role of Al in the formation of secondary Ni

precipitates on pyrophyllite, gibbsite, talc, and amorphous silica: A DRS study. *Geochim. Cosmochim. Acta* **1999**, *63*, 3193-3203.

(11) Wang, X. S.; Huang, J.; Hu, H. Q.; Wang, J.; Qin, Y. Determination of kinetic and equilibrium parameters of the batch adsorption of Ni(II) from aqueous solutions by Na-mordenite. *J. Hazard. Mat.* **2007**, *142*, 468-476.
